# Exploring optimal HAART adherence rates in Ethiopian adults: a systematic review and meta-analysis

**DOI:** 10.3389/fpubh.2024.1390901

**Published:** 2024-10-14

**Authors:** Mengistie Yirsaw Gobezie, Nuhamin Alemayehu Tesfaye, Tewodros Solomon, Mulat Belete Demessie, Teklehaimanot Fentie Wendie, Getachew Tadesse, Tesfaye Dessale Kassa, Fentaw Tadese Berhe, Minimize Hassen

**Affiliations:** ^1^Department of Clinical Pharmacy, School of Pharmacy, College of Medicine and Health Sciences, Wollo University, Dessie, Ethiopia; ^2^Department of Statistics, College of Natural Sciences, Wollo University, Dessie, Ethiopia; ^3^Department of Clinical Pharmacy, College of Health Sciences, Mekelle University, Mekelle, Ethiopia; ^4^Department of Epidemiology and Biostatistics, School of Public Health, College of Medicine and Health Sciences, Wollo University, Dessie, Ethiopia; ^5^Public Health & Economics Modeling Group, School of Medicine & Dentistry, Griffith University, Gold Coast, QLD, Australia

**Keywords:** adherence, antiretroviral therapy, adult, HIV medication, meta-analysis, Ethiopia

## Abstract

**Background:**

Optimal medication adherence is vital for the successful implementation of highly active antiretroviral therapy (HAART) in managing HIV infection. Global efforts aim to minimize the burden of antimicrobial resistance (AMR), including HIV-associated drug resistance.

**Methods:**

This systematic review and meta-analysis followed PRISMA guidelines and searched multiple databases for eligible studies published until July 10, 2023. Eligible studies focused on Ethiopians receiving HAART, reported the prevalence of optimal adherence, and used appropriate assessment tools. Quality of included studies was assessed using JBI checklists A weighted inverse variance random-effects model was applied to calculate the pooled prevalence.

**Results:**

Our meta-analysis aimed to determine the pooled prevalence of optimum Highly Active Antiretroviral Therapy (HAART) adherence among HIV-positive adults in Ethiopia and explore variations based on assessment methods, recall periods, and regional factors. The estimated national pooled prevalence of optimal HAART adherence was 79% (95% CI: 74–83, *I*^2^ = 98.1%; *p*-value < 0.001). Assessment methods revealed a prevalence of 64% (95% CI: 54–73) using structured assessment and 82% (95% CI: 78–86) with self-reporting. Optimum adherence varied based on recall periods, ranging from 78 to 85% with self-reporting. Heterogeneity analysis indicated substantial variation (*I*^2^ = 98.1%; *p*-value < 0.001), addressed through subgroup analysis, sensitivity analysis, and univariate meta-regression. Subgroup analysis based on region identified varying prevalence: SNNPR (83%), Oromia (81%), Amhara (79%), and Addis Ababa (74%). Considering the 2018 guideline revision, year-based subgroup analysis showed a prevalence of 78% and 78% before and after 2018, respectively. Sensitivity analysis demonstrated the stability of results, with excluded studies having a minimal impact. Publication bias analysis indicated an absence of bias, as evidenced by a non-significant Egger's regression test (*p*-value = 0.002) and no adjustment in trim and fill analysis.

**Conclusions:**

The estimated overall prevalence of optimal adherence was 79%, indicating a substantial level of adherence to HAART in the Ethiopian context. The study identified variations in adherence levels based on assessment methods and recall periods, highlighting the importance of considering these factors in evaluating adherence rates. These insights contribute valuable information for policymakers, healthcare practitioners, and researchers working toward enhancing HAART adherence in Ethiopia.

**Systematic review registration:**

https://www.crd.york.ac.uk/prospero/display_record.php?RecordID=459679

## Introduction

Globally, according to a recent World Health Organization (WHO) report, 39 million people were living with HIV, 1.3 million had new HIV infections, and 630,000 faced AIDS-related deaths in 2022. Out of these, 37.5 million people living with HIV and 540,000 HIV/AIDS deaths were exhibited among the adult population (≥15 years of age) (1). Continent-wise, the African Region appears to be most gravely impacted, with roughly 1 in every 25 adults (3.2%) living with HIV and constituting more than two-thirds of the people living with HIV (2). Ethiopia being one of the erstwhile countries in sub-Saharan Africa massively shares the burden. Roughly, 11,000 Ethiopian adults aged 15 and above died because of AIDS-related illnesses in 2020 (3).

Following the issuance of the 2002 National Antiretroviral Drugs Supply (ARVS) and use policy, Ethiopia was among the first African countries to acquaint antiretroviral therapy (ART) in selected health facilities in 2003 (1). ART is a combination of different types of antiretroviral medications used for lifetime treatment of HIV to boost the quality of life of patients by increasing levels of CD4 cells and minimizing viral load (4). Sustainable and optimum adherence to therapy is a major factor that determines the success of HAART (5, 6). This is mainly because poor adherence has a significant impact on the emergence of virologic failure, viral resistance, and grievous adverse health outcomes such as substance abuse, depression, and the spread of infections (7–9).

Although HAART plays a paramount role in improving the lives of HIV-positive patients, HIV-related morbidity and mortality occurred substantially even in the presence of HAART regimens (10, 11). One of the decisive factors that determines the efficacy and enduringness of HAART regimens is medication adherence (12). Though the minimum value for optimum HAART adherence is not well-delineated, at least ≥ 95% adherence is indicated for optimal therapeutic effect (13, 14). The most frequently reported barriers to optimum HAART adherence are attributed to socio-demographic, behavioral, clinical, and health-system related factors (4, 15, 16).

Apparently, both national and international systematic review and meta-analysis studies (17–20) have been conducted among the children population. In Ethiopia, numerous observational studies (3, 16, 21) have been conducted related to the prevalence of adherence to HAART and its associated factors among HIV-infected adults. However, massive discrepancies were reported amidst these studies even within the same geographical setting, across regions, at similar and different time intervals. For instance, the magnitude of optimum adherence to HAART among HIV-infected adults varied from 72.4 to 94.3% in Ethiopia (3). Consequently, the pooled prevalence of optimum HAART adherence among HIV-infected adults remains ill-defined. Hence, this systematic review and meta-analysis study aimed to determine the national pooled prevalence of optimum adherence to HAART among adult HIV patients in Ethiopia.

## Methods

### Study protocol

Records identification, titles, and abstract screening along with full-text eligibility evaluation for the final analysis was done in accordance with The Preferred Reporting Items for Systematic Reviews and Meta-analysis (PRISMA) algorithm (22). The study protocol was successfully registered at PROSPERO with a reference number: CRD 42023459679.

### Databases and search strategy

A stringent inclusive literature search was performed to retrieve studies reporting the prevalence of optimum HAART adherence and associated predictors of HIV-positive adults in Ethiopia. Both electronic and gray literature searches were conducted in a systematic manner. A comprehensive search was carried out in PubMed, Google Scholar, Hinari, SCOPUS, and EMBASE electronic databases up to 10 July 2023. The search was centered on studies/articles published in the English language with a reported prevalence of optimal HAART adherence among Ethiopians living with HIV. The following terms and/or phrases were employed: “Adherence^*^,” “Nonadherence^*^,” “Noncompliance^*^,” “Non-Adherence^*^,” “Non Adherence^*^,” “poor adherence,” “good adherence,” “Persistence^*^,” Compliance^*^,” “Non-Compliance^*^,” “Non Compliance^*^,” “ART,” “Highly Active Antiretroviral Therapy,” “HAART,” “Combination Antiretroviral Therapy,” “Antiretroviral Therapies.” “Antiretroviral Therapy” AND “Ethiopia”. Search strings were employed using “AND” and “OR” Boolean operators. Moreover, the proceedings of professional associations and university repositories were filtered. Furthermore, a direct Google search and reference tracing were also executed by deploying the bibliographies of the identified studies to incorporate further relevant studies missed while digging into the electronic databases.

### Inclusion and exclusion criteria

The studies were incorporated if they met the following inclusion criteria: studies conducted on adult Ethiopians who are on HAART; observational studies, including cross-sectional, cohort, and case-control studies; studies that reported prevalence of optimum adherence; studies which clearly delineate the measurement tool used for assessment of adherence and operationally defined optimum adherence as patients who took 95% or more of the recommended dose during the specified recall period; studies conducted in Ethiopia; studies published only in the English language. Included studies in this meta-analysis used two types of adherence measurement tools to examine optimum HAART adherence: the self-report method and the structured assessment method. Qualitative studies and citations without full text and studies on the sphere of adherence to HAART prophylaxis were precluded.

### Study selection and quality assessment

Endnote version 20 reference manager (23) was deployed to remove duplicated studies. Two independent reviewers (MY and TD) autonomously screened the titles and abstracts to consider the articles in the full-text review. Two investigators (MH and TS) assessed the quality of the studies by using Joanna Briggs Institute's (JBI) critical appraisal checklist for studies reporting prevalence data (24). The following items were used to appraise the selected studies: (1) appropriateness of the sampling frame to address the target population, (2) appropriateness of participants sampling technique and adequacy of sample size, (3) detailed description of study subjects and setting, (4) sufficient analysis of the data and validity and reliability of HAART adherence assessment methods, (5) appropriateness of statistical analysis used and adequacy of response rate. The disagreements were resolved by mutual consensus via blunt joint discussion with a third reviewer. Studies scored five and above out of nine were considered low risk.

### Data extraction

A Microsoft Excel format was synthesized for data abstraction. Two independent reviewers (MY and MH) extracted the data. The procedure was repeated whenever a discrepancy appeared. Data pertaining to the first author and year of publication, regions where studies were conducted, study design, study setting, sample size, total number of adherents, assessment tools used, number of days used as a recall period for calculation of adherence level, and the thresholds used for definition of optimum adherence were extracted. In all included studies, structured questionnaires, and self-report techniques were used as assessment methods for measuring adherence levels. Accordingly, we have thematized studies based on their assessment tools as self-report and structured questionnaire methods. Furthermore, studies that use self-report as a measuring tool used different recall time periods prior to the interviews and thus were further classified based on their duration of recall period as 7, 15, and 30 days. Hence, data regarding the level of adherence was collected in four groups in this meta-analysis.

### Data analysis

A weighted inverse variance random-effects model (25) was employed to estimate the pooled prevalence of optimal HAART adherence. Subgroup analysis was conducted to adjust for variation in the pooled estimates based on the specific region where the studies were conducted and the year of publication. Due to the inherent variability in the accuracy of adherence measurements, which can be affected by both the assessment methods and the recall periods used, a subgroup analysis was conducted, this allowed us to account for potential differences in adherence estimates based on how the data was collected and the length of the recall period.

Heterogeneity among the included studies was examined using *I*^2^ statistic by considering 25, 50, and 75% as low, moderate, and high heterogeneity (26). To ascertain the risk of publication bias, a Funnel plot and Egger's regression test were performed (27). A sensitivity analysis was conducted to ascertain the stability of the summary estimate after the omission of individual studies. STATA version 17 statistical software (28) was used to carry out this meta-analysis.

### Outcome of interest

In this meta-analysis, our main objective is to assess the prevalence of optimal adherence to Highly Active Antiretroviral Therapy (HAART) among Ethiopian adults living with HIV. For this review, optimal adherence is operationally defined as consuming 100% of the recommended dose for recall periods of 15 days or less. For recall periods of 30 days and above, optimal adherence was defined as patients taking 95% or more of the recommended dose during this timeframe. The population of interest includes HIV-positive adults undergoing highly active antiretroviral therapy (HAART) in Ethiopia. Our primary outcome is the determination of optimal adherence to HAART. The study designs considered encompass observational studies that detail adherence to ART among HIV-positive individuals in Ethiopia, utilizing a variety of adherence assessment methods. In this study, we categorized adherence assessment methods into structured assessment and self-report methods.

## Result

### Characteristics of included studies

**Figure 1:**
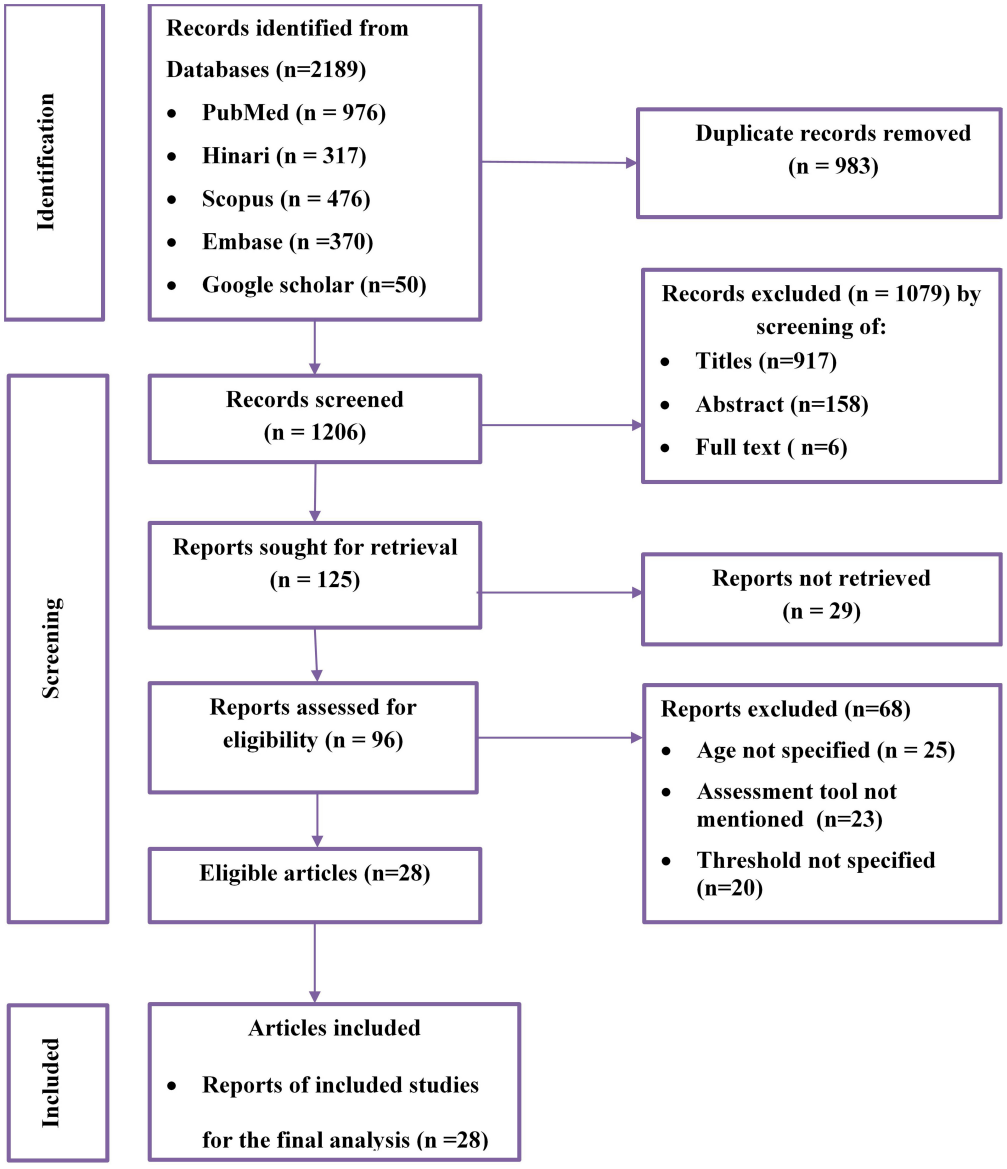
Flow diagram of the included studies for the systematic review and meta-analysis of optimum adherence to HAART in Ethiopia.

A total of 2,189 potential studies were identified; 976 articles from PubMed, 317 articles from Hinari (research4life), 370 from EMBASE, 476 articles from Scopus, and 350 articles from other sources. Figure 1 shows the results of the search and reasons of exclusion during the study selection process. A total of 28 articles were included to assess the prevalence of optimal adherence to HAART in Ethiopia. The included studies were published between the years 2007 and 2022. Of the included studies, 7 of them were published after 2018, a year in which the national ART guideline was updated. A cross-sectional study design was used for all included studies.

Eight studies were conducted in the Amhara region (29–36), seven in Oromia (37–44), five in SNNPR (21, 45–48), five in Addis Ababa (49–53), one in Harare (3), one in Harari and Dire Dawa city (54) and one in Benishangul Gumuz (55). Twenty-three studies reported the prevalence of optimal adherence based on self-report method (21, 29, 30, 32–42, 44–48, 50, 51, 53, 54, 56) while six studies (3, 31, 43, 49, 52, 55) reported via structured adherence assessment method. Among 23 studies that assessed optimal adherence based on the self-report method: seven studies used 7 days (33, 38, 44, 48, 50, 53, 54), three studies used 15 days (39, 42, 47), and eleven studies used 30 days (21, 29, 30, 32, 34, 36, 37, 40, 41, 46, 51, 56) recall period. A total of 11,388 adults living with HIV and on HAART participated in the included studies. Table 1 depicts the characteristics of the included studies.

**Table 1:** General characteristics of the included studies (*n* = 28).

**Authors**	**Study year**	**Region**	**Study design**	**Study setting**	**Sample size**	**No of adherence**	**Tools used**	**Recall period**	**Quality score**
Amberbir et al.	2007	Oromia	Cross sectional	Hospitals	400	384	Self-report	7 days	Low risk
Beyene et al.	2007	SNNPR	Cross sectional	Hospital	422	393	Self-report	15 days	Low risk
Tessema et al.	2008	Amhara	Cross sectional	Hospitals	504	417	Self-report	1 day	Low risk
Tiruneh et al.	2008	Addis Ababa	Cross-sectional	Hospital &HC	102	90	Self-report	7 days	Low risk
Tiyou et al.	2009	Oromia	Cross sectional	Hospital	319	303	Self-report	7 days	Low risk
Tilahun et al.	2010	Addis Ababa	Cross sectional	Hospital	1,722	1,034	SA	NA	Low risk
Negash et al.	2011	Addis Ababa	Cross sectional	Hospital	355	261	Self-report	7 days	Low risk
Berhe et al.	2020	SNNPR	Cross sectional	Hospital &HC	417	284	Self-report	30 days	Low risk
ketema et al.	2012	Amhara	Cross sectional	Hospital &HC	422	403	Self-report	30 days	Low risk
Letta et al.	2012	H&D	Cross sectional	Hospital &HC	620	527	Self-report	7 days	Low risk
Tsega et al.	2014	Amhara	Cross-sectional	Hospital	351	284	Self-report	30 days	Low risk
Jima et al.	2015	Oromia	Cross-sectional	Hospital	160	137	Self-report	15 days	Low risk
Mola et al.	2015	Amhara	Cross sectional	Hospital	440	389	Self-report	30 days	Low risk
Ejigu et al.	2017	Oromia	Cross sectional	Hospital	284	230	Self-report	30 days	Low risk
legesse et al.	2017	Amhara	Cross sectional	Health center	418	300	Self-report	7 days	Low risk
Abebe et al.	2018	Addis Ababa	Cross sectional	Hospital &HC	368	302	SA	NA	Low risk
Aferu et al.	2018	SNNPR	Cross sectional	Hospital	103	68	Self-report	90 days	Low risk
Damtie et al.	2018	Amhara	Cross sectional	Hospital &HC	292	144	SA	7 days	Low risk
Dorsisa et al.	2018	Oromia	Cross-sectional	Hospital	303	164	Self-report	15 days	Low risk
Rike et al.	2018	SNNPR	Cross sectional	Hospital	370	320	Self-report	7 days	Low risk
Tegegne et al.	2018	Harari	Cross sectional	Hospitals	501	332	SA	NA	Low risk
Abadiga et al.	2019	Oromia	Cross sectional	Hospital	305	223	Self-report	30 days	Low risk
Asrat et al..	2019	Amhara	Cross-sectional	Hospital	393	322	Self-report	30 days	Low risk
Aychiluhm et al.	2019	Amhara	Cross-sectional	Hospital &HC	310	256	Self-report	30 days	Low risk
Nigusso et al.	2017	BG	Cross sectional	Hospital &HC	390	235	SA	NA	Low risk
Angelo et al.	2020	SNNPR	Cross sectional	Hospital	329	274	Self-report	30 days	Low risk
Girma et al.	2022	Oromia	Cross sectional	Hospitals	412	344	Self-report	30 days	Low risk
Tadesse et al.	2022	Addis Ababa	Cross sectional	Hospital	388	261	Self-report	30 days	Low risk

SNNPR, Southern nation nationality and people region; H&D, Harari and Dire Dawa; BG, Benishangul-Gumuz; HC, Health center; NA, Not applicable; SA, Structured Assessment.

### Quality of the included studies

All of the included studies reporting prevalence data were assessed using the JBI critical appraisal checklist (24). The JBI quality appraisal checklist assessment indicated that none of the incorporated studies were pitiful in quality and precluded from the meta-analysis (Table 1).

### Meta-analysis

#### Pooled prevalence of optimum HAART adherence in Ethiopia

**Figure 2:**
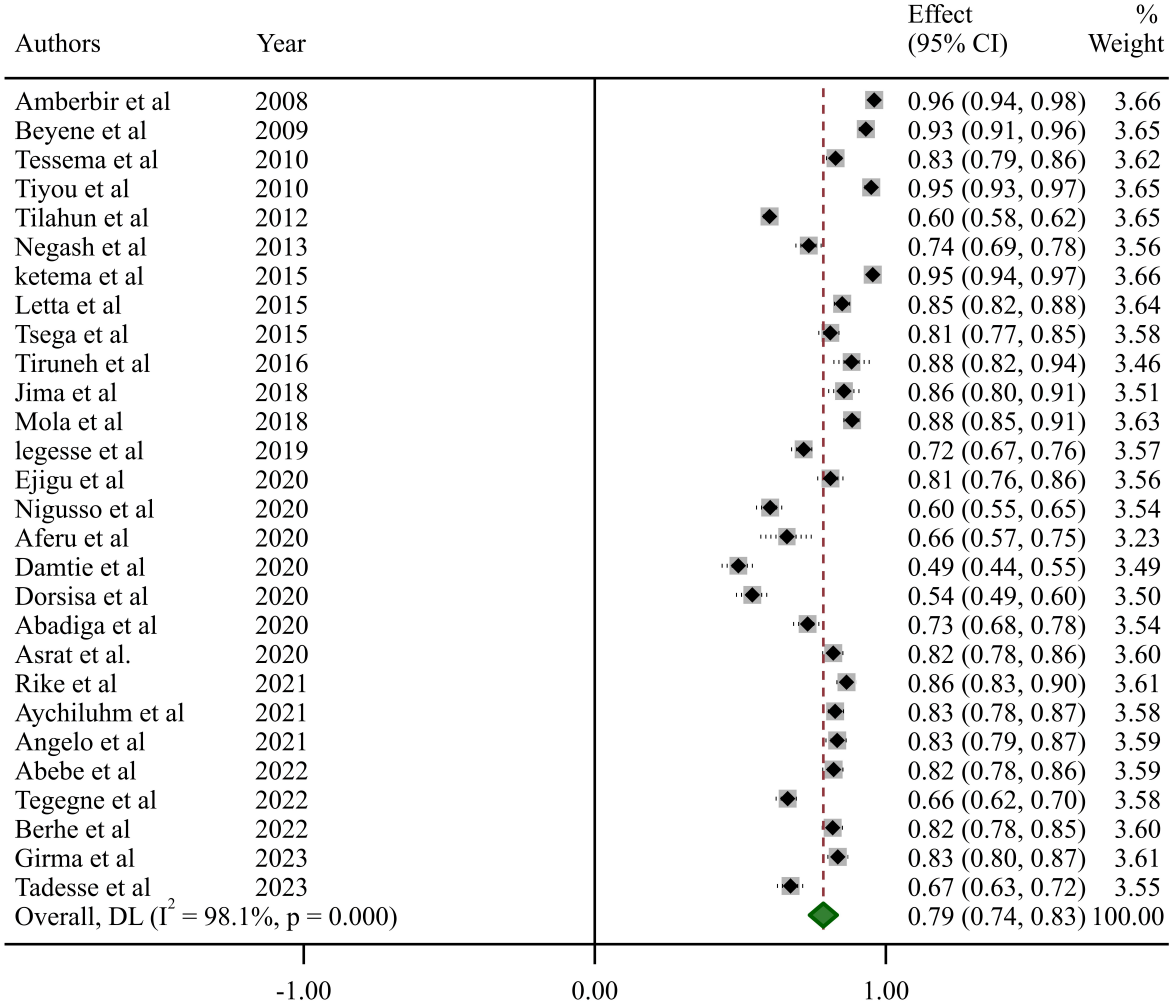
Pooled estimate of the prevalence of optimum adherence of HAART in Ethiopia.

In this systematic review and meta-analysis, the estimated national pooled prevalence of optimal HAART adherence among HIV-positive adults in Ethiopia was 79% (95% CI: 74–83, *I*^2^ = 98.1%; *p-*value < 0.001) (Figure 2).

#### Heterogeneity analysis

**Figure 3:**
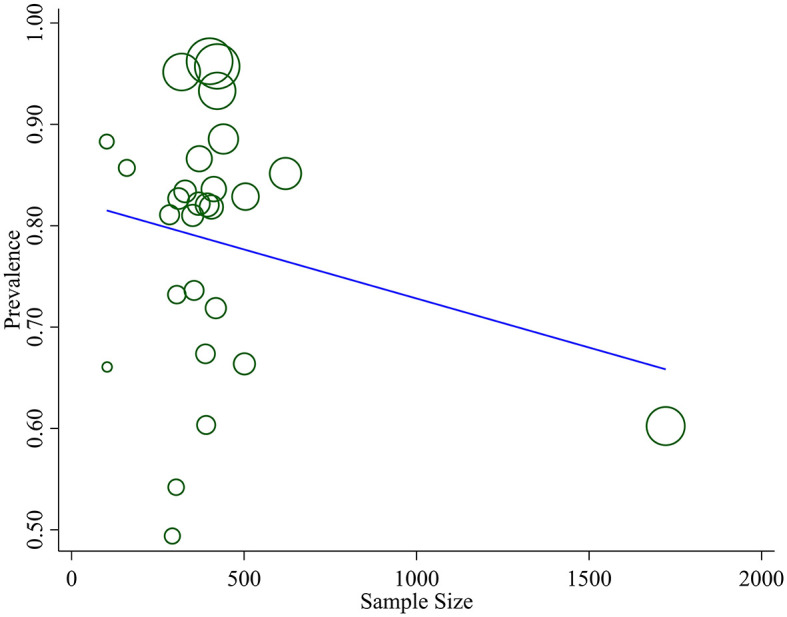
Meta regression of prevalence of optimum HAART adherence and sample size.

Studies incorporated for the analysis demonstrated massive heterogeneity (*I*^2^ = 98.1%; *p-*value < 0.001), which was not adequately addressed with a weighted inverse variance random-effects model. For further analysis of this heterogeneity, we used a funnel plot as a subjective assessment and conducted subgroup analysis, sensitivity analysis, and univariate meta-regression for objective assessment of the etiologies of heterogeneity (Figure 3; Table 2).

**Table 2:** Meta regression of prevalence of optimum HAART adherence and sample size.

Meta-regression					Number of obs = 28	
REML estimate of between-study variance					tau^2^ = 0.01476	
% residual variation due to heterogeneity					*I*-squared_res = 97.45%	
Proportion of between-study variance explained with Knapp-Hartung modification					Adj R-squared = 1.55%	
***P***	**Coefficient**	**Std. err**.	***t***	***P*** > **|*****t*****|**	**(95% conf. interval)**	
Sample size	−0.0000968	0.0000845	−1.15	0.263	−0.0002704	0.0000769
_cons	0.8249324	0.041796	19.74	0.000	0.7390195	0.9108452

#### Subgroup analysis

Subgroup analysis based on the region where studies were conducted revealed that the highest prevalence of optimum HAART adherence was in SNNPR at 83% (95% CI: 77–90, *I*^2^ = 92.8%; *p* < 0.001), while the lowest was in Addis Ababa at 74% (95% CI: 63–85, *I*^2^ = 97.2%; *p* < 0.001). Additionally, subgroup analysis based on the study years was conducted using 2018 as a dichotomization point, given that it marked the revision of the Ethiopian ART guideline and the full implementation of the test-and-treat approach (57). The prevalence of optimum HAART adherence among adults before 2018 was 78% (95% CI: 72–84, *I*^2^ = 98.5%; *p* < 0.001) and 79% (95% CI: 75–83, *I*^2^ = 86.6%; *p* < 0.001) after 2018.

Subgroup analysis based on study methods revealed that the prevalence of optimum HAART adherence was 64% (95% CI: 54–73, *I*^2^ = 96.7%; *p* < 0.001) when assessed with structured assessment methods, and 82% (95% CI: 78–86, *I*^2^ = 96.4%; *p* < 0.001) when examined with the self-report method To further address the impact of recall periods on adherence levels, we analyzed optimal HAART adherence within subgroups categorized by different recall periods. The national pooled prevalence of optimum HAART adherence at 7, 15, and 30 days prior to an interview was 85% (95% CI: 79–92, *I*^2^ = 96.8%; *p* < 0.001), 78% (95% CI: 55–100, *I*^2^ = 98.7%; *p* < 0.001), and 82% (95% CI: 77–87, *I*^2^ = 95.1%; *p* < 0.001), respectively (Table 3).

**Table 3:** Subgroup analysis of the prevalence of optimum HAART adherence based on different variables.

**Groups**	**Subgroups**		**Adherence level % (95% CI)**	***I*^2^ %**	***p*-values**
Regions	Amhara		79 (71–89)	97.7	<0.001
	Addis Ababa		74 (63–85)	97.2	<0.001
	Oromia		81 (72–90)	97.9	<0.001
	SNNPR		83 (77–90)	92.8	<0.001
Year of study	Before 2018		78 (72–84)	98.5	<0.001
	After 2018		79 (75–83)	86.6	<0.001
Assessment methods	Structured		64 (54–73)	96.7	<0.001
	Self-report		82 (78–86)	96.4	<0.001
	Recall periods	7 Days	85 (79–92)	96.8	<0.001
		15 Days	78 (55–100)	98.7	<0.001
		30 Days	82 (77–87)	95.1	<0.001

SNNPR, Southern Nations Nationalities and Peoples' Region.

#### Sensitivity analysis

We did the sensitivity analysis of adherence to HAART by applying a random effects model (Table 4). Excluded studies with a smaller sample size resulted in a slight difference in the pooled prevalence of optimal HAART adherence, which did not significantly affect the stability of the summary result.

**Table 4:** Sensitivity analysis of studies included for estimation of optimum adherence to HAART in Ethiopia.

**References**	**Estimate**	**(95% conf. interval)**	
Amberbir et al. (38)	0.77859718	0.7312572	0.82593715
Beyene et al. (47)	0.77964324	0.73102403	0.82826245
Tessema et al. (35)	0.78356844	0.73459595	0.83254087
Tiruneh and Wilson (53)	0.78171062	0.73353064	0.82989067
Tiyou et al. (44)	0.77896589	0.73086339	0.82706845
Tilahun et al. (52)	0.79269087	0.75092882	0.83445299
Negash and Ehlers (50)	0.7870388	0.7388702	0.8352074
Ketema and Shewangizaw Weret (32)	0.77877253	0.73108047	0.82646459
Letta et al. (54)	0.78268999	0.73336279	0.83201718
Tsega et al. (36)	0.78428257	0.73569298	0.83287215
Jima and Tatiparthi (42)	0.78259975	0.73428851	0.83091098
Molla et al. (34)	0.7814284	0.73243642	0.83042043
Ejigu et al. (40)	0.78426719	0.7357977	0.83273661
Legesse and Reta (33)	0.78770131	0.73964292	0.83575976
Abebe and Tegegne (49)	0.78384626	0.73517358	0.83251894
Aferu et al. (45)	0.78938758	0.74160993	0.83716518
Damtie and Tadese (31)	0.79587787	0.75001866	0.84173715
Dorsisa et al. (39)	0.79413128	0.74768996	0.8405726
Rike et al. (48)	0.78217638	0.7333594	0.83099335
Tegegne et al. (3)	0.78978819	0.74236161	0.83721477
Abadiga et al. (37)	0.78717947	0.73906696	0.83529204
Asrat et al. (29)	0.78389108	0.73517591	0.83260626
Aychiluhm et al. (30)	0.78366536	0.73508799	0.83224279
Nugus and Irena (43)	0.79197812	0.74510795	0.83884829
Angelo and Alemayehu (21)	0.78339708	0.73475653	0.83203763
Berhe et al. (56)	0.78396648	0.73523813	0.83269477
Girma et al. (41)	0.78329825	0.73447692	0.83211958
Tadesse et al. (51)	0.78937012	0.74171817	0.83702207
Combined	0.78520637	0.73811713	0.8322956

#### Publication bias

**Figure 4:**
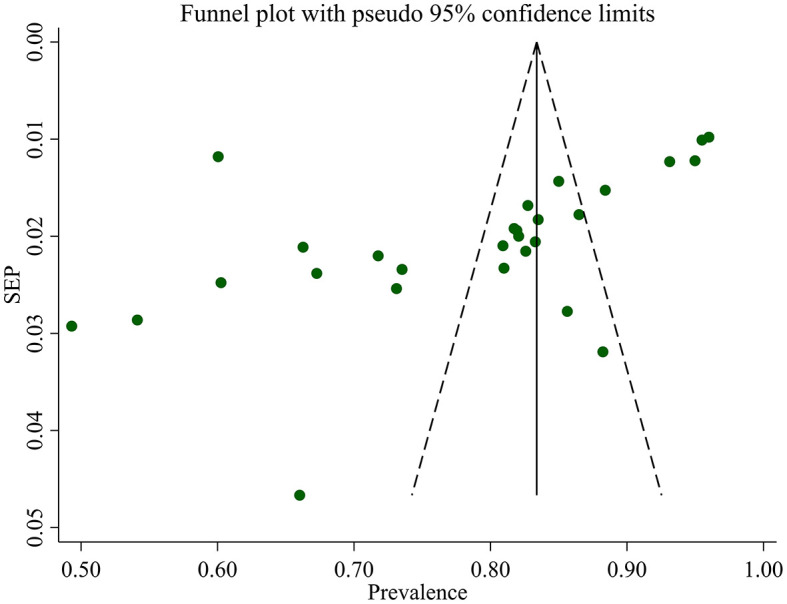
Funnel plot of optimum HAART adherence in Ethiopia.

We assessed publication bias both subjectively and objectively. Subjectively, we observed the funnel plot (Figure 4), and objectively, we conducted Egger's regression test, which resulted in a *p*-value of 0.002. Additionally, we performed a trim and fill analysis (Table 5), which neither added nor removed studies, indicating the absence of missed small studies and, ultimately, the absence of publication bias.

**Table 5:** Trim and fill analysis of optimum adherence to HAART in Ethiopia.

**Method**	**Pooled est**	**95% CI**		**Asymptotic**		**No. of studies**
		**Lower**	**Upper**	**z_value**	***p*** **_value**	
Fixed	0.834	0.827	0.840	253.259	0.000	28
Random	0.785	0.738	0.832	32.682	0.000	

Test for heterogeneity: Q = 1,390.435 on 27 degree of freedom (*P* = 0.000), Moment-based estimate of between studies variance = 0.016.

## Discussion

The present systematic review and meta-analysis were conducted to estimate the national pooled prevalence of optimal HAART adherence among HIV-infected adults in Ethiopia. The finding of this study is indispensable to crafting policies and contriving public health interventions to trim HIV-associated mortality and morbidity. Accordingly, the pooled prevalence of optimal HAART adherence among HIV-positive adults in Ethiopia was 79% (95% CI 74–83%; *I*^2^ = 98.1%). The finding of this meta-analysis was comparable to a study conducted in China (77.6%) (58), Africa (77%) (2), and Latin American, and Caribbean countries (70%) (59) and recently reported finding in Ethiopia (60). However, it was higher than a globally meta-analyzed report (62%) (61), studies done in India (70%) (19), and West Africa (42%) (62) as well as recently published single studies in Ethiopia (63, 64), but lower compared to studies done in South India (90.9%) (18), and US (86%) (65). The discrepancies amidst these studies might be ascribed to the variations in socio-demographic characteristics, study population, study design, healthcare systems, recall period, and the adherence report method. Perhaps, the figure is indicative of promising progress which is not consonant with the developed nation's misconception that sub-optimal therapy is apparent in the African regions.

The optimum HAART adherence prevalence via structured assessment method and self-report method were 64 and 82%, respectively. Although there is no masterpiece assessment tool for determining HAART adherence, studies (66, 67) spotlighted that the self-report method may lead to an overestimation of the pooled prevalence of true adherence owing to its subjective nature as compared to the more objective tools. This might be a possible concern engrossed to our study too. A precisely 18% difference is noted in terms of the proportion of HAART adherence between these adherence measurement tools.

In this meta-analysis, a lower pooled prevalence of HAART adherence in Ethiopia was reported among the studies published before 2018 (78%), as compared to those published after 2018 (79%). The possible reason for this could be better provision of targeted adherence measures, improved ART medication availability and accessibility, and implementation of the updated guideline treatment recommendation in the actual clinical setting. It is apparent that medical science is dynamic in its nature necessitating updated knowledge, skills, and attitudes.

The subgroup analysis showed that HAART adherence among adults was highest in the SNNPR region (83%) compared to other regions, with the lowest adherence observed in Addis Ababa (74%). This incongruity might be owing to the difference in the health care system and clinical setting, socio-economic assets, attitude, and awareness of caregivers about HAART regimens. Moreover, it could also be attributed to the regions' varied beliefs about the merit of HAART, religious practices, political affiliations, and traditional medicine usage practice. This finding is different from a similar study (17) in Ethiopia done on children which exhibited the highest prevalence in the Amhara region (93.4%), Addis Ababa (90.1%), Oromia region (73.04%), and Tigray region (87.3%), respectively.

The findings in this study reveal variations in adherence prevalence based on assessment methods, recall periods, and regional factors, shedding light on the multifaceted nature of adherence evaluation.

Assessment methods play a crucial role in determining reported adherence rates. Our study indicates that when assessed using structured adherence assessment questionnaires, the optimum HAART adherence prevalence was lower at 64%. In contrast, self-reporting yielded a higher prevalence of 82%. This discrepancy emphasizes the impact of the chosen assessment tool on reported adherence rates, suggesting that the methodological approach can influence the interpretation of adherence levels.

Furthermore, the influence of recall periods on reported adherence rates is evident in our study. The prevalence of optimum adherence assessed by the self-report method varied from 78 to 85%, depending on the recall period used (07, 15, or 30 days prior to an interview). This finding highlights the importance of considering the timeframe for assessing adherence, as it can significantly impact the reported prevalence.

The observed massive heterogeneity (*I*^2^ = 98.1%; *p*-value <0.001) among the studies necessitated additional analyses. While a weighted inverse variance random-effects model was insufficient to address the heterogeneity, subjective assessments through funnel plots and objective analyses such as subgroup analysis, sensitivity analysis, and univariate meta-regression were conducted. These approaches aimed to explore potential sources of heterogeneity, providing a more nuanced understanding of the variability in reported adherence rates.

Subgroup analysis based on regional factors identified variations in adherence prevalence among different regions of Ethiopia. Higher adherence rates were observed in SNNPR, Oromia, and Amhara compared to Addis Ababa, suggesting potential regional disparities in healthcare infrastructure and support systems. Additionally, the year-based subgroup analysis, considering the implementation of the test and treat approach in 2018, showed a slight increase in adherence prevalence after this pivotal year.

Sensitivity analysis, which involved excluding studies with smaller sample sizes, demonstrated the stability of the overall results, with minimal impact on the pooled prevalence of optimal HAART adherence. Publication bias analysis, conducted through Egger's regression test and trim and fill analysis, indicated the absence of bias, reinforcing the reliability of our findings.

## Strength and limitation

The study employed a comprehensive and rigorous search strategy to identify all possibly available eligible primary studies. This approach enhances the inclusivity of the analysis, ensuring that a wide range of relevant studies was considered and it also comprehensively assessed optimal adherence to HAART based on various methods, including both self-reporting and structured adherence assessment questionnaires. This approach allows for a more nuanced exploration of adherence levels, capturing the diverse ways in which adherence is measured in the included studies. The study addressed various factors that may affect the prevalence of optimal adherence to HAART through extensive sub-group analysis. This approach enables the exploration of heterogeneity among studies, allowing for a more detailed examination of regional and temporal variations. However, it is not devoid of limitations, the primary limitation of this study is the absence of more reliable biological methods for assessing adherence in the included primary studies. The reliance on self-reporting and structured assessments, with varying recall periods across the included primary studies, may impact the homogeneity of the studies. While this is common in adherence research and introduces a subjective element along with the potential for recall bias, we attempted to address this by providing operational definitions. Additionally, several studies in our review employed non-probability sampling methods, which could introduce selection bias and ultimately affect the accuracy of the pooled estimate.

## Conclusion

This systematic review and meta-analysis revealed that overall HAART adherence in Ethiopian adults appears in good progress and is consistent with findings noted from other developing countries. However, it still requires a lot of work to bring the adherence level to its theoretically presumed value (100%). The lowest prevalence of optimum HAART adherence is observed in Addis Ababa, the capital city of Ethiopia. Patient-specific interventional measures should be rigorously performed by underpinning potential predictors of HAART adherence to make it more promising.

## Data Availability

The original contributions presented in the study are included in the article/supplementary material, further inquiries can be directed to the corresponding author.
